# Catastrophic Aortoenteric Fistula Due to an Eroding Bare Metal Duodenal Stent

**DOI:** 10.7759/cureus.16115

**Published:** 2021-07-02

**Authors:** Ryan Stuart, Harold Duarte, Aamir Pervez, Lex P Leonhardt

**Affiliations:** 1 Internal Medicine, Kettering Medical Center, Kettering, USA

**Keywords:** aorto-enetric fistula, duodenal stent, gastro intestinal bleeding, metastatic rcc, duodenal ulceration, massive hemoptysis, massive blood transfusion, hematochezia

## Abstract

Deployment of bare metal duodenal stents for individuals with gastric outlet obstructions (GOOs) is a well-characterized measure to improve the quality of life. However, these interventions are palliative in nature and are associated with known complications. We present an unfortunate case of a previously not well described, albeit not surprising, a complication of stent placement. The individual underwent duodenal stent placement due to obstructive metastatic disease and subsequently presented for gastrointestinal (GI) bleed. It was determined that an aortoduodenal fistula acutely developed and, despite heroic efforts, the patient ultimately expired.

## Introduction

Gastric outlet obstructions (GOOs) are a well-documented sequela of primary and metastatic disease encroaching on the stomach, duodenum, and subsequent components of the proximal digestive tract [1]. Palliative application of endoscopically placed metal stents has demonstrated improved ability for patients to maintain oral intake [2-4]. It has been reported that following stent placement, up to 90% of patients have relief of symptoms that may be associated with gastrointestinal (GI) obstructions [5-6]. Enteral stenting, however, does come with significant risks. Complications can range from mild to severe and life-threatening [7-9]. The most common complications are migration of covered stents and tumor ingrowth in bare stents, with bleeding and perforation being the least common (<1%) [8]. Here, therefore, we present a case of a patient presenting with a primary aortoenteric fistula (AEF) caused by an eroding duodenal stent that was originally placed for palliative treatment of malignant GOO.

## Case presentation

A 58-year-old male presented to the emergency department (ED) after a single episode of hematemesis, denying melena or hematochezia at that time. He had an extensive past medical history, pertinent for renal cell carcinoma (RCC) diagnosed at age 38 with subsequent right nephrectomy. Fifteen years later he was found to have pancreatic metastasis with a confirmed biopsy of RCC. Ultimately a high-grade GOO developed secondary to increasing pancreatic metastases with resulting mechanical compression of the duodenum. As a palliative maneuver, an uncovered self-expanding bare metal duodenal stent (120 mm x 22 mm) was placed with no intraoperative or immediate postoperative complications. He had significant improvement in symptoms and was able to tolerate enteral intake.

Four months after stent placement, he presented with an episode of hematemesis as described in the presentation above. CT scan of the abdomen/pelvis without IV or oral contrast at the time of admission demonstrated increased abdominal tumor burden, but no intra-abdominal bleeding. He was admitted and underwent esophagogastroduodenoscopy (EGD) which demonstrated multiple duodenal ulcers, suspected to be malignant in origin at the time of the procedure. Stenosis of the distal end of the duodenal stent was noted, however, the scope was able to be advanced into the third part of the duodenum despite this. None of the duodenal ulcers were noted to have signs of recent or active bleeding, therefore, no periprocedural intervention was undertaken.

**Figure 1:**
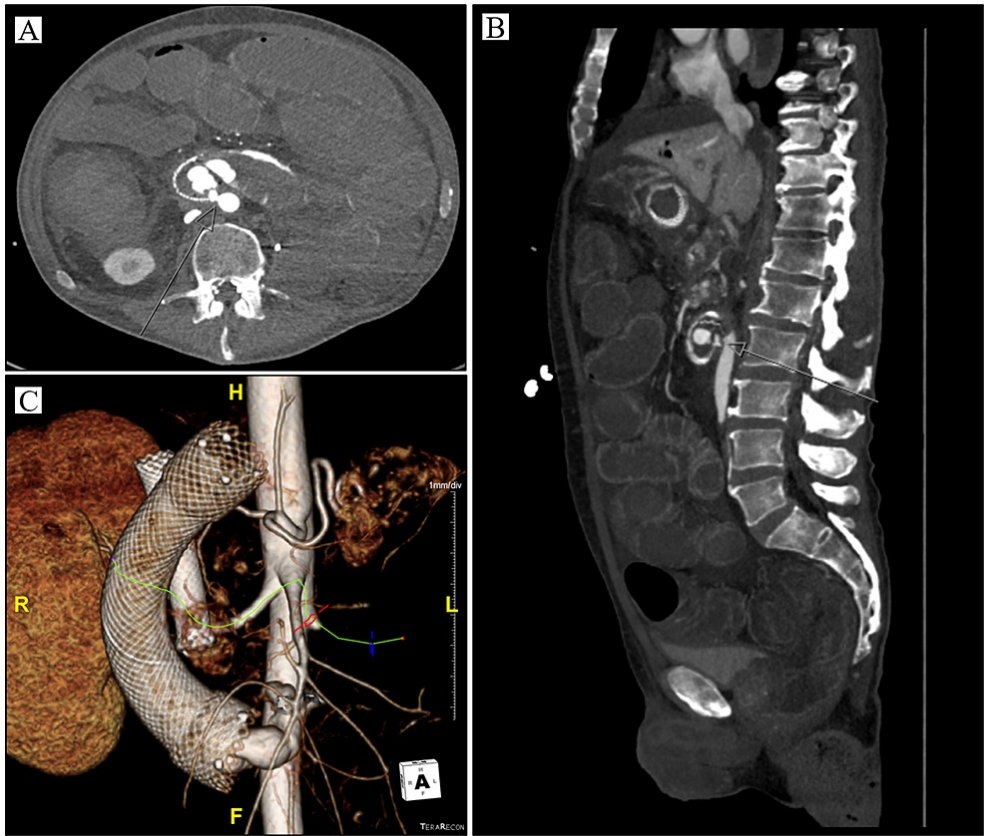
Abdominal imaging following stabilization. CT of the abdomen and pelvis with IV contrast visualizing AEF in the transverse plane (A), sagittal plane (B), and with 3D reconstruction (C). AEF, aortoenteric fistula

Post-procedure, the patient was initially hemodynamically stable, however, that evening he began having large volume hematochezia with intermittent hematemesis. He developed profound hypotension consistent with hemorrhagic shock and was transferred to the ICU. While receiving ICU level care, he was found to have progressive abdominal distension with continuous high output hematochezia. Massive transfusion protocol was activated, he was intubated for airway protection, and was transiently stabilized. He was transported for emergent imaging. CT angiography demonstrated an aortoduodenal fistula, suspected to be secondary to wall perforation by the distal end of the duodenal stent (Figure 1). Perihepatic and intra-abdominal free fluid, likely extravasated blood from the fistula, was also newly identified. The patient's hemodynamic status remained tenuous while receiving continuous volume replacement. A collaborative discussion was had with all consulting services including gastroenterology, interventional radiology, vascular surgery, and general surgery throughout the case. Ultimately it was determined that any procedural intervention would be futile and, following a discussion, the patient's family changed his code status to comfort care. Unfortunately, the patient expired shortly thereafter. 

## Discussion

Malignant gastric outlet obstruction (GOO) is an unfortunate but not unusual sequelae of many neoplastic processes. This is a complication that develops in almost 20% of patients with primary pancreatic, gastric or duodenal carcinoma, especially with metastatic disease [10]. Tumor metastasis to the pancreas is a rare occurrence, presenting in approximately 2%-5% of cases, and usually found to be secondary to RCC, melanoma, colorectal carcinoma, breast carcinoma, or various sarcomas [7]. Presenting symptoms of malignant GOO often include nausea, vomiting, abdominal distension, or abdominal pain. This is often a life-changing complication for patients as it leads to recurrent dehydration, progressive malnutrition, and overall diminished quality of life.

Palliative treatment for malignant GOO focuses on the re-establishment of GI patency. In order to achieve this goal, there have been historically two therapeutic approaches for symptomatic improvement, surgical gastrojejunostomy (GJ), and enteral stenting. Surgical GJ focuses on creating an anastomosis between the stomach and proximal loop of the jejunum. Enteral stenting by the placement of endoscopic self-expanding metal stents (SEMS) is another option that has gained popularity over the years. While many variations of enteral stents with different structures are now available, the overall association between stent type and clinical outcomes is still poorly understood [11].

While SEMS is the preferred treatment approach for many patients due to their rapid symptomatic relief and clinical success rate, these stents are not without risk of complications. Placement of the stent itself can be challenging due to anatomic variations and the difficulty of passing a guidewire through a severely narrowed section of stenosis [7]. Other reported challenges for successful stent placement include stent migration following placement and stent impaction into the small bowel wall. Complications for SEMS are found to range between 11% and 43% and are classified as immediate (within one day), early (from 96 h to two weeks), or late. Our patient’s aorto-duodenal fistula would certainly be deemed a late complication of stent placement. While an uncommon sequela of duodenal stent placement, prompt recognition of fistula formation is key in managing this catastrophic complication.

Aortoenteric fistula (AEF) is a rare but serious complication. Secondary AEF occurs more often following aortic intervention in comparison to enteric intervention, but even following aortic repair, this complication is rare and occurs in approximately 0.3%-2% of cases [12]. AEF is not uncommonly preceded by a brisk, self-limiting event GI bleed, or a “Herald bleed” as seen in our patient. While primary AEF has been reported in patients without prior history of aortic/enteric intervention or recent trauma, it is less common than secondary AEF. CT imaging with IV contrast is considered the most appropriate initial diagnostic technique in suspected AEF due to the shorter time to completion, increased likelihood of possible complications from endoscopic imaging, and increased hemodynamic instability of these patients [13]. Effective treatment focuses on an endovascular repair with subsequent surgical repair, if possible. Unfortunately, due to the emergent and progressive nature of this complication, outcomes remain very poor with extremely high mortality rates regardless of intervention, as was the case with our patient.

## Conclusions

This case describes a unique and previously not well-described mechanism of injury by bare metal duodenal stents, specifically in the formation of an AEF. Our case illustrates how important it is to strongly consider AEF in any patient with a history of enteric stenting or previous aortic reconstruction surgery who presents with massive GI hemorrhage. High suspicion with prompt work-up can lead to a shorter time to treatment and possibly improved mortality outcomes.
